# Diffusion Mechanism in Running-Water and CFD-DEM Numerical Simulation of Expandable Particulate Grouting Material

**DOI:** 10.3390/ma18071681

**Published:** 2025-04-07

**Authors:** Zhipeng Zhang, Chenyang Ma, Chen Zhao, Zhuo Zheng, Wei Li, Rentai Liu, Xiuhao Li, Hongyan Wang

**Affiliations:** 1 School of Civil Engineering, Institute of Geotechnical and Underground Engineering, Shandong University, Jinan 250061, China; 202315052@mail.sdu.edu.cn (Z.Z.); 202222201112@mail.sdu.edu.cn (C.Z.); zhengzhuo22@sdjzu.edu.cn (Z.Z.); rentailiu@163.com (R.L.); lixiuhao@mail.sdu.edu.cn (X.L.); 2 State Key Laboratory of Intelligent Construction and Healthy Operation and Maintenance of Deep Underground Engineering, China University of Mining and Technology, Xuzhou 221116, China; tbh259@cumt.edu.cn; 3 Jinan Urban Construction Group Co., Ltd., Jinan 250013, China; liyong250014@163.com

**Keywords:** CFD-DEM, numerical model, grout material, expandable particulate

## Abstract

In order to study the diffusion and sealing mechanism of an innovative grouted material tentatively called “expandable particulate grout material”, the diffusion process was simulated by the numerical method of CFD-DEM coupling. A numerical model was established for a grouting process in an individual fracture based on the basic physical parameters of expandable particles. The numerical model of the expandable particulate slurry flow was established. The interaction between particles and water in different conditions, such as different grouting times, different volume fractions of the particle, and different velocities, was investigated. The differences in the diffusion process and in the running-water sealing mechanism of expandable particles, cement slurry, and cement-sodium silicate slurry in the crack (in a, in b, and in c) were analyzed. The influence of expandable particles on the streamline of the grout and the drag force in the interaction process under the fracture were analyzed. This is summarized The influence of the velocity ratio of grout to water on different physical quantities, such as diffusion opening degree, diffusion velocity, and diffusion distance, was summarized. It is of significant theoretical and practical value to further develop and improve the grouting technology.

## 1. Introduction

There are plenty of natural defects such as fracture structural planes in the rock mass. During the bearing process, the instability of the fractured rock mass will start to break from weak places such as the fracture structural plane, from part to the whole, and finally destroy the entire rock mass. Grouting is an important means to ensure safe construction [1,2,3,4]. In recent years, through the cross innovation with computational fluid dynamics, materials science, and other emerging disciplines, the theoretical system of grouting technology has achieved significant development, and a variety of anti-dispersion grouting materials in running water and corresponding diffusion theories have been developed [5,6,7].

In terms of materials science: related scholars have developed a large number of grouting materials for different engineering problems. According to the gel particles, it can be divided into granular slurry and chemical slurry. Among them, the granular slurry mainly includes portland cement, sulfoaluminate cement, and cement-sodium silicate. The chemical slurry mainly includes polyurethane, epoxy resin, polyacrylic acid, lignin, and various types of modified grouting material on this basis [8,9,10,11,12,13]. However, for the sealing of rapid water inrush (>1 m/s), the current traditional Portland cement grouting materials have a long setting time (6–8 h), so the effect is poor. The cement-sodium silicate slurry is highly sensitive to setting time, and it is easy to block the drilling and damage to the grouting machine. Acrylate and lignin grouting materials are highly toxic and cause environmental damage. Therefore, how to resist the adverse effects of the scouring of running water on the grouting material, contrary, to developing a grouting material that relies on the running-water scouring force to achieve effective slurry transport, diffusion, and sealing is the key to the treatment of water inrush on the fractured structural surface in the water-rich zone.

In terms of computational fluid dynamics: in order to carry out more in-depth calculation and evaluation research on fractured rock mass grouting, a stepwise numerical calculation method is proposed, which takes into account the viscosity change caused by chemical reaction and the decrease in soil porosity caused by the deposition of grout particles and describes the process of cement-based grouting material entering porous media [14]. In addition, different grout constitutive equations, such as Newtonian fluid, Bingham fluid, and power law fluid, are used in the theoretical analysis. Many numerical methods, such as FEM/VOF and time-step calculation methods, are used to describe the grouting process [15,16,17,18]. According to the morphological characteristics of the slurry-water interface in the process of slurry diffusion, theoretical and experimental research are carried out, and the relevant mathematical characterization methods are established [19]. Carried out the fracture dynamic water grouting test and analyzed the influence relationship between the pressure field, the grouting method, and the effect [20]. A lot of work has also been conducted on the fissure running-water grouting test system, and the theoretical model research of dynamic water diffusion has been carried out [21]. Furthermore, many scholars have used the CFD-DEM [22,23,24] numerical calculation method to simulate particle flow and present a coupled model based on computational fluid dynamics (CFD) and discrete element method (DEM) to simulate sediment bed erosion by viscous shear flow at various shear flow velocities [21]. Present, the field in geotechnical engineering has made a series of beneficial progress in studying particle-loaded flows through CFD-DEM coupling.

Aiming at the problem that the slurry is easy to disperse and lose its coagulation ability under running water, Li et al.’s team developed a new type of expandable particle grout material, which is suitable for large-flow water inrush sealing [25,26]. The material is composed of special gel particles and carrier fluid, which are configured in a certain proportion. After entering the groundwater environment, the gel particles use the capillary action of the internal cross-linking network of the material to quickly absorb water and expand (up to 300 times their own volume); the free water is bound inside the material so as to slow down the flow rate of groundwater or directly transform the groundwater environment into a static state. Because the expansion of the expandable particle grouting material does not depend on the chemical reaction, the material will not fail due to the action of flowing water, avoiding the damage of high-pressure and large-flow water inrush to the material reaction process. Its physical and chemical properties have unique advantages in many grouting materials [21]. However, the research on this type of grouting material is very limited, and there is a lack of corresponding grout rheological model and flow calculation theory. Regarding the coupling of particle migration and expansion during dynamic water grouting, no relevant research has been carried out yet. When applying the theory of fluid mechanics to the calculation of slurry flow, it is necessary to follow the assumption of a constant flow pattern. The sealing problem of grouting under running water involves the whole process of gradual transformation of slurry from fluid to solid, which leads to difficulties in calculation and analysis. In this paper, the CFD-DEM method is used to study the slurry diffusion under running water and the plugging mechanism of the expandable particulate grouting material. The structure of this article is as follows. First, the expandable particle grouting material was used as the research object to test its working performance. Then, the CFD-DEM coupling principle is analyzed. Finally, a numerical simulation was carried out for the problem of grouting with dynamic water in a single plate fissure, and a numerical model of expandable particle grouting material flow was established. The interaction between particles and fluid and the flow field changes during particle expansion and migration are studied in detail. The differences between the expandable particle grouting material and the cement grouting material and cement-sodium silicate grouting material under running water in the fissure grouting material diffusion law and sealing mechanism have been analyzed, compared, and revealed.

## 2. Physical and Chemical Properties of Expandable Particles Grouting Material

### 2.1. Introduction to Expandable Particulate Material

**Figure 1:**
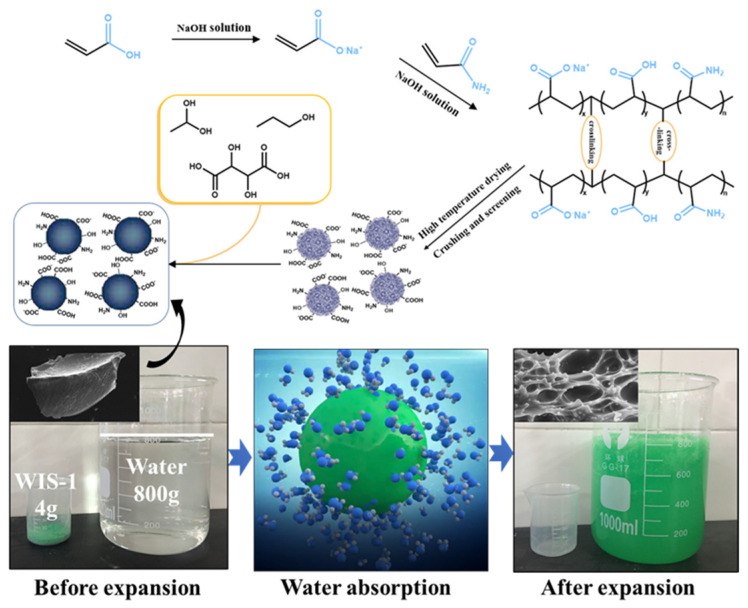
Gel particle synthesis principle, expansion effect, and water absorption mechanism.

Expandable granular material is a kind of ultra-high-expansion granular grouting material suitable for water inrush sealing of karst pipelines and consists of polyacrylic resin with water absorption and expansion characteristics. Specifically, the material is composed of gel particles and carrier fluid. Gel particles are a kind of high molecular polymer formed by cross-linking copolymerization of acrylic acid and acrylamide (see Figure 1) [25,26]. This polymer contains a large number of strong hydrophilic groups (see the molecular synthesis diagram of the material); the molecular structure shows a three-dimensional crosslinked network (see Figure 1, the bottom three-dimensional mesh structure schematic); through the hydration effect, it can quickly absorb hundreds of times its own mass of water; the morphology of the gel (see Figure 1, the bottom before and after the expansion of the material) is gel-like. Gel particles have the advantages of strong water-absorbing capacity, fast water absorption, and strong water retention capacity and are non-toxic and odorless. Gel particles have the appearance of solid powder and cannot be used directly in the grouting process, so they have to be used together with the carrier liquid. The carrier liquid consists of ethanol and glycerin, which play the role of suspending and transporting the gel particles. When the gel particles are exposed to water, the water molecules are hydrated with hydrophilic groups through hydrogen bonding, and the hydrophilic groups begin to dissociate, causing osmotic pressure to build up in the material and water molecules to penetrate into the mesh structure. Therefore, the water absorption process of the gel particles is a physical process and is not affected by scouring. During the grouting process, it is not necessary for the material to resist the disturbance of flowing water. Instead, the material is expected to diffuse with the groundwater, absorbing it and slowing its flow as it migrates until it is sealed.

### 2.2. Material Performance

The size of the gel particle has an important influence on the expansion performance. To this end, >100 mesh, 100–50 mesh, and 50–30 mesh gel particles (2 g) are wrapped in tea bags, put into water, and taken out every 30 s. A centrifuge (speed 1000 r/min, time 1 min) was used to remove residual moisture. By measuring the mass change of the gel particles before and after water absorption, the expansion ratio can be calculated. The calculation formula is shown in Equation (1).

(1) $$S_{w}=\frac{m_{i}-m_{0}}{m_{0}}$$

**Figure 2:**
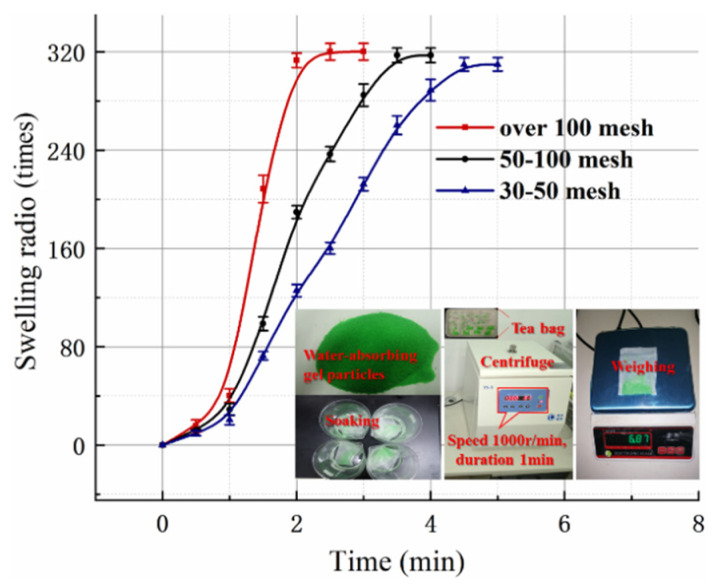
The swelling ratio of gel particles with different meshes.

In the formula, m_0_ and m*_i_* are the mass of the gel particle before and after swelling; S_W_ stands for Expansion Rate. The expansion ratio of gel particles with different meshes is measured as shown in Figure 2. As mentioned, gel particles have superior water absorption properties; the expansion ratio was as high as 320, and the particle size has little effect on the expansion ratio. In addition, the smaller the particle, the larger the specific surface area and the faster the water absorption rate. The time required for the super absorbent polymer (subsequently abbreviated to SAP) >100 mesh particles to fully expand was half that of the 30–50 mesh particles upon water exposure. Therefore, the expansion rate can be adjusted according to the engineering requirements.

## 3. CFD-DEM Coupling

### 3.1. CFD-DEM Coupling Principle

#### 3.1.1. Grid Division and Calculation of Flow Field Information

In order to ensure the accuracy of calculation, when discretizing the flow field, the divided fluid grid unit should not be too coarse. At the same time, the length of the grid unit should be large enough relative to the particle size to ensure that the particle unit has a sufficiently long time interval in each grid unit. For this reason, the mesh element length should meet the following constraints when building the model:

(2) $$6r<\Delta x_{cfd}<\frac{1}{5}d_{\min}$$

(3) $$\Delta x_{cfd}>3\left|u\right|t_{c}$$

In the Formulas (2) and (3), $∆x_{cfd}$ is the length of the grid unit, $d_{min}$ is the minimum side length dimension of the calculation area, ***u*** is the gel particle velocity, and $t_{c}$ is the fluid-structure coupling calculation time step.

Since the stiffness of the gel particles will gradually decrease after absorbing water; there will be a large overlap when the particles collide with the wall boundary. When the length of the overlapping part exceeds the radius of the particle, the particle will pass through the wall and escape. Therefore, in the calculation process, the stiffness of the particles needs to meet the following constraints (4):

(4) $$\frac{2}{3}\pi r^{3}\rho_{p}{\left|u_{\max}\right|}^{2}<\frac{k_{n}r^{2}}{8}$$

where, $\rho_{p}$ is the particle density and $k_{n}$ is the normal contact stiffness of the particle.

#### 3.1.2. Calculation Method of Particle-Fluid Interaction

The fluid force on the gel particles is determined by calculating the following formula:

(5) $$\frac{\partial u}{\partial t}=\frac{f_{m}+f_{l}}{m}+g$$

(6) $$\frac{\partial \omega }{\partial t}=\frac{M}{I}$$

In the Formulas (5) and (6), ***u*** is the velocity of the gel particles, ***m*** is the mass of gel particles, $f_{l}$ is the total force of the fluid on the gel particles, and $f_{m}$ is all forces on the particles except fluid force (such as contact force between particles, gravity, etc.), ***g*** is the acceleration of gravity, ω is the angular velocity of the gel particles, **M** is the momentum received by the particles, and ***I*** is the moment of inertia of the particles. The specific expression of $f_{l}$ is:

(7) $$f_{l}=f_{drag}+\frac{4}{3}\pi r^{3}\left(\nabla p-\rho_{l}g\right)$$

From Equation (7), shows that the force of the fluid on the particles consists of two parts. The first term is the drag force $f_{drag}$ caused by the velocity difference between the fluid and the particles, and the second term represents the fluid pressure caused by the pressure gradient. In order to obtain the specific expression of $f_{drag}$, the velocity difference between the fluid and the particles must be determined firstly. If ignoring the influence of gel particles on fluid flow and the interaction between particles, the drag force on a single particle in the flow field can be expressed as:

(8) $$f_{0}=\frac{1}{2}C_{d}\rho_{l}\pi r^{2}\left|u-v\right|\left(u-v\right)$$

In the Formula (8), $C_{d}$ is the drag force coefficient, $\rho_{l}$ is the fluid density, ***r*** is the particle radius, ***v*** is the fluid velocity, and ***u*** is the particle velocity.

The specific expression of the drag coefficient $C_{d}$ is shown in the Formula (9).

(9) Cd=0.63+4.8Rep2

${Re}_{p}$ is the Reynolds number of the suspension shown in the Formula (10).

(10) Rep=2ρlru−vμl

As the particles continuously absorb water and expand during the slurry flow process, the volume occupied by the particles in the fluid unit continues to increase, and the fluid porosity continues to decrease. At the same time, as the particle spacing keeps getting smaller, the interaction between the particles keeps increasing, and the Reynolds number of the suspension keeps changing. Therefore, it is necessary to add a correction factor to the fluid drag force of a single particle. The drag force of any particle in the slurry flow process can be expressed as the Formula (11).

(11) $$f_{drag}=f_{0}\varepsilon^{-\chi }$$

ε is the instantaneous porosity of the fluid unit. χ is an empirical parameter, and specifically expressed as (12):

(12) χ=3.7−0.65exp−1.5−log10Rep2/2

At the same time, it should be pointed out that within the same fluid unit, the fluid velocity at different spatial positions is the same, but there are certain differences in the flow velocity in different fluid units. Since particles will continue to traverse from one fluid unit to other units during the migration process, the drag force components of different units on the particle need to be calculated according to the volume percentage of the overlap between the particle and different units. At the same time, when calculating the resultant force, the point of action of each component force is located at the center of the particle, and the drag force will not produce a rotational effect on the particle. Therefore, the resultant force of the drag force exerted by different fluid units on the gel particles can be expressed as (13):

(13) $$f_{b}=\frac{\Sigma_{j}f_{drag}^{j}}{V}$$

### 3.2. Calculation Model

The shape of the fracture is a regular cuboid, which conforms to the basic assumption of a single flat crack: the inclination angle of the crack is set to zero; that is, the influence of gravity on the spread of grout and the flow of groundwater is ignored. The upper and lower walls of the crack are parallel plates with smooth surfaces, and the crack opening at any point inside the crack is a constant value.

**Figure 3:**
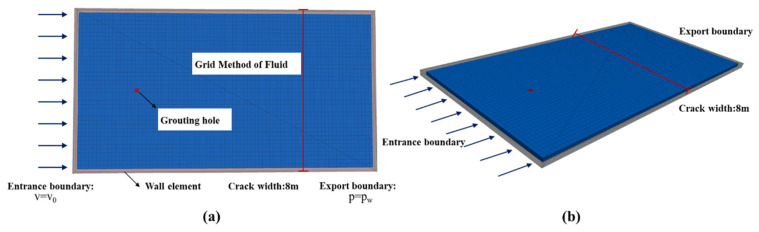
Geometric model and boundary condition. (**a**) Geometric model, (**b**) Boundary conditions.

The geometric model and boundary conditions of the fracture grouting diffusion simulation are shown in Figure 3. Set the crack aperture to 0.2 dm and the distance between the two sides of the crack to 80 dm. The fluid grid size is a rectangular parallelepiped with a length of 2 dm, a width of 2 dm, and a height of 0.2 dm. Set the groundwater on the inlet boundary as a vertical inflow, and set it as a constant velocity boundary with 1 dm/s. Set the groundwater on the outlet boundary to be in a vertical outflow state, and its groundwater pressure is zero. The boundary on both sides of the fracture is set as an impervious boundary; that is, there is no flow in the direction perpendicular to the boundary. The grouting hole is located on the bisector of the boundary on both sides, and the grouting flow is 2 L/s, 4 L/s, 6 L/s, 8 L/s, and 10 L/s. Assuming that the shape of the expandable particle material is a regular sphere with the same particle size, the calculation uses a spherical element to approximate the expanded particles and simulate the mechanical behavior during the flow process. The initial content of expanded particles in the slurry is 1%. In the actual calculation, the distance between the entrance boundary and the grouting hole is three times the maximum counter-current diffusion of the slurry, and the distance between the export boundary and the grouting hole is 50 dm longer than the maximum along-water diffusion distance of the slurry. The various parameters of fluid and particle properties in the calculation are shown in Table 1.

## 4. Analysis on Slurry Diffusion and Sealing Mechanism of Fracture

### 4.1. Diffusion Law of Fissure Grouting in Running Water

#### 4.1.1. Diffusion Morphology of Different Types of Slurry

**Figure 4:**
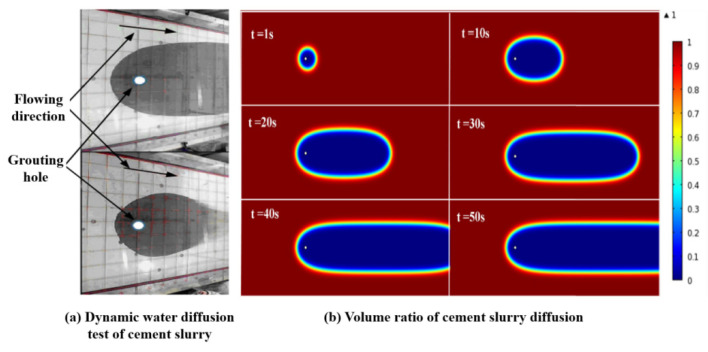
(**a**) The diffusion pattern and (**b**) numerical simulation results of cement slurry in running water.

In previous studies, a series of experiments and numerical studies have been carried out on the running-water diffusion law of common cement slurry and cement sodium silicate slurry [27,28]. The diffusion pattern and numerical simulation results of cement slurry in a running-water test are shown in Figure 4. In the early stage, the diffusion shape of cement slurry is an asymmetric ellipse, and in the later stage, the shape of diffusion is oval in the counter-current direction, and the diffusion range along water is obviously larger than that of counter to the water. It is considered that the flow plays a dominant role in the initial stage of diffusion due to the small amount of slurry entering. With the increase in grouting time, the slurry begins to deposit continuously along the flow direction from the grouting hole, and the slurry diffusion shape gradually changes from a closed ellipse shape to a U shape. After reaching stability, with the increase in grouting time, the diffusion pattern basically does not change. It can be inferred that the diffusion of slurry is blocked by groundwater and more slurry is transported downstream due to the scouring effect of water flow.

Some literature also points out that under dynamic water conditions, the early diffusion of cement slurry is easily affected by water flow, and the diffusion range along the water flow is larger than that against the water flow, which is consistent with the results of this study and verifies the dominant role of water flow in the early stage of diffusion. The inference in this study is that the diffusion morphology of the slurry is affected by the blockage of the reverse water flow and the transportation along the water flow, lacking consideration for the dynamic changes in the physical and chemical properties of the slurry itself. For example, the dynamic changes of the slurry under dynamic water conditions can also affect the diffusion morphology, which has not been fully reflected in previous studies. [29,30] In addition, this study suggests that even if the diffusion morphology remains basically unchanged after stabilization, the internal structure of the slurry may still undergo slow adjustment, thereby affecting the overall diffusion effect.

**Figure 5:**
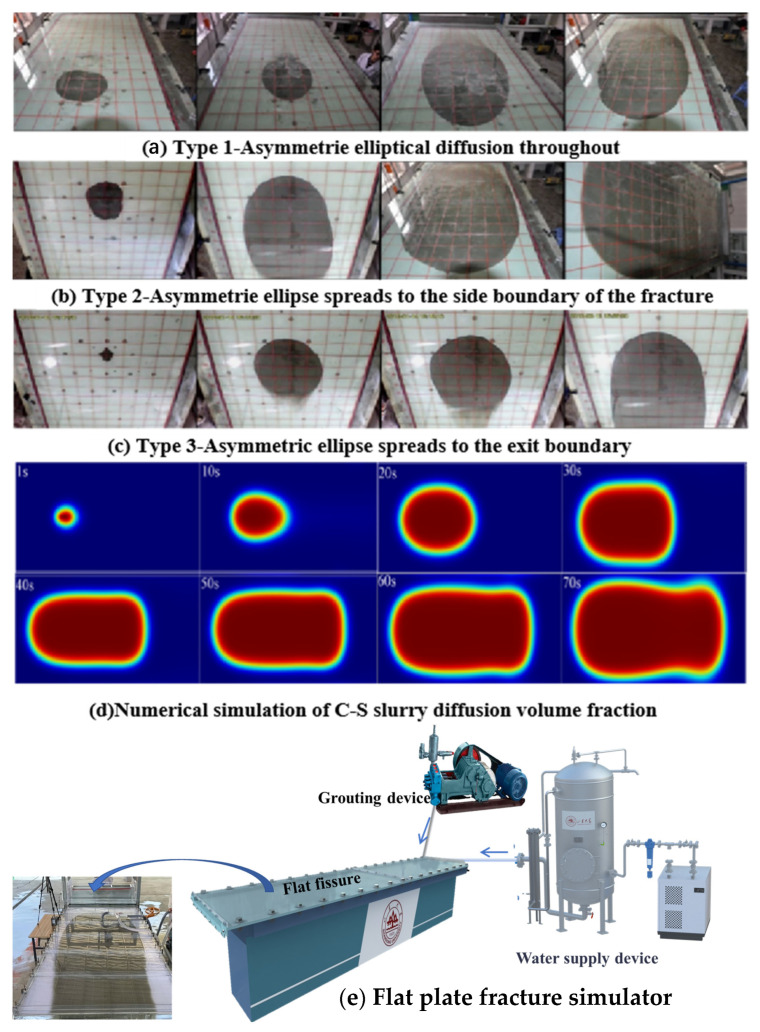
(**a**–**c**) Experimental morphology of cement-sodium silicate slurry in running water, (**d**) is the numerical simulation morphology, and (**e**) Flat plate fracture simulator.

The experimental morphology and the numerical simulation morphology of cement-sodium silicate slurry under running-water conditions are shown in Figure 5. During the diffusion of quick-setting slurry, the slurry diffusion trace forms an asymmetric ellipse that changes over time, with variations influenced by the model boundary. When the slurry solidifies faster and the velocity of flow is not large, the grout reaches the final pressure of grouting and does not spread to the boundary of the model and forms the asymmetric elliptical diffusion in the whole process. See Figure 5a, when the slurry injection rate is fast, the slurry curing time is moderate, and the hydrodynamic velocity is small, the slurry diffusion will reach the solid wall boundary, move along the solid wall boundary to the outflow boundary, form an asymmetric ellipse, and diffuse to the fracture side boundary, as shown in Figure 5b. When the slurry injection rate is slow, the hydrodynamic flow rate is fast, and the slurry solidification time is long, the slurry reaches the outlet boundary, and in this process, the slurry does not diffuse to the solid wall boundary, forming an asymmetric ellipse, which diffuses to the export boundary, as shown in Figure 5c.

**Figure 6:**
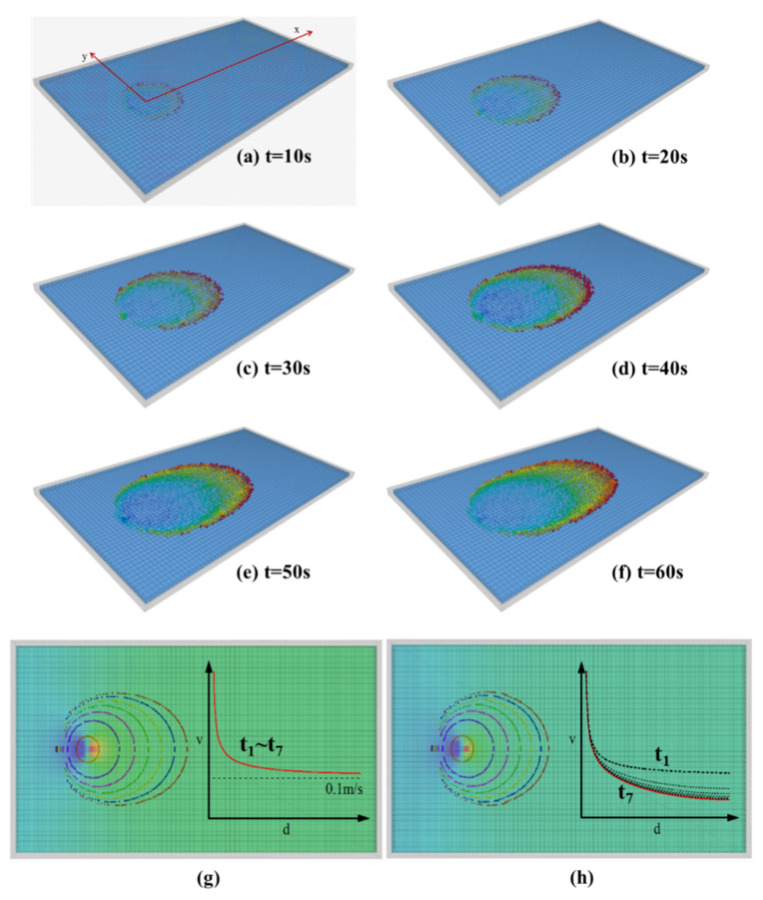
(**a**–**f**) Diffusion range of expanded particle slurry and the expanded state of particles with grouting time. (**g**) The diffusion profile of the gel particles when the flow field in the fracture remains unchanged and the velocity along the water diffusion direction on the central axis of the fracture changes with time. (**h**) The diffusion profile and velocity change obtained when the groundwater velocity gradually decreases under the influence of the gel particles.

Figure 6 shows the diffusion range of expandable particle slurry and the front profile of the slurry diffusion at different times. Observing the slurry diffusion in Figure 6, the slurry diffusion shape is approximately circular at the initial time of grouting, and then it is approximately elliptical. The reason is as follows: at first, the flow of gel particles is mainly controlled by the injection velocity at the grouting hole, and the initial velocity of slurry entering into the fracture near the grouting hole is much higher than that of groundwater, so the slurry diffusion along all directions is approximately equal. With the increase in grouting time, the diffusion range of slurry gradually increases. The influence of grouting pressure on the particles near the slurry front decreases gradually, and the influence of groundwater flow on the slurry diffusion increases gradually. The slurry mainly diffuses along the direction of groundwater flow, and the diffusion form gradually changes from round to oval. If the slurry diffusion shape is regarded as an irregular ellipse, the major axis of the ellipse coincides with the central axis. The ellipse is symmetrically distributed along its long axis; the velocity, pressure, and expansion volume of the slurry are symmetrically distributed on both sides. While on both sides of the short axis, the physical field information is not symmetrical. Under the influence of groundwater flow, the position of the short axis moves backward along the direction of groundwater flow with the increase in grouting time.

It can be seen from Figure 6g,h that the gel particles entering the fracture at the same time are always in a closed curve during the diffusion process; that is, the particles that enter the fracture first are always in the outermost layer of the slurry diffusion range. At the same time, the water absorption time of the gel particles entering into the fracture in the early stage is longer. Therefore, the closer to the outside of the slurry diffusion range, the larger the particle expansion volume, the higher the resistance in the process of gel particle migration, and the faster the diffusion rate attenuation. Comparing the particle diffusion profiles at different times, it can be found that in Figure 6g, the interval length of the profile gradually decreases from the grouting hole to the outside and tends to be stable. The reason is that with the increase in particle diffusion range, the velocity of gel particles finally approaches the velocity of groundwater. In Figure 6h, when the effect of particle volume expansion on the velocity is considered, the velocity at any point in the fracture decreases continuously with time. The particle outside the diffusion region has a higher degree of water absorption ratio, which leads to a greater attenuation of the diffusion rate, so the contour interval length decreases continuously from the inside to the outside.

By comparing the dynamic water grouting of expanded particle slurry, cement slurry, and cement-sodium silicate slurry, it can be found that the diffusion patterns of the three have similar rules. Among them, cement slurry diffuses in the model under the joint action of hydraulic transportation, scouring, and concentration gradient. Affected by factors such as grouting rate, dynamic water flow rate, slurry solidification rate, and other factors, the diffusion process of cement sodium silicate slurry has different forms. According to the shape characteristics of the irregular ellipse in the process of slurry diffusion, the space-time change equation describing the slurry diffusion shape was established by the curve-fitting method. However, under the influence of many factors, such as fracture shape, groundwater velocity, grouting rate, and slurry viscosity, it is difficult to establish a theoretical model of slurry diffusion covering all factors. The CFD-DEM method is used to calculate the slurry diffusion form more accurately, which can directly reflect the process of particle water absorption and expansion and fully consider the interaction between fluid and suspended particles so as to avoid the difficulty of defining the slurry macro viscosity. The calculation results are more in line with reality and reflect the rheological characteristics of expanded particle slurry more truly.

#### 4.1.2. The Effect of Expandable Particles on Fluid Flow Field

**Figure 7:**
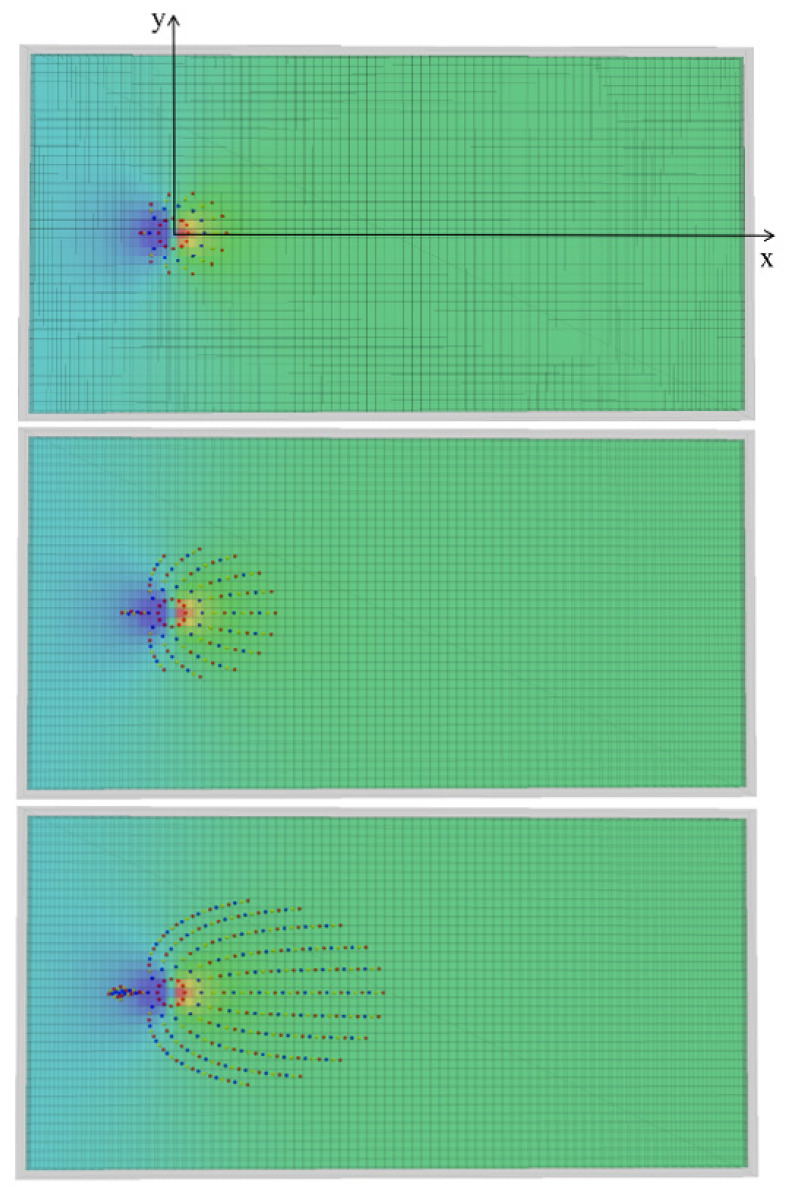
The transport trajectory of gel particles at different times under constant flow conditions.

Take the flow field in the fissure at the initial moment of grouting as the benchmark. Calculate the particle movement on the circle with the grouting hole as the center and the radius is 0.3 m. In this way, the particle migration trajectory at different moments and the streamline distribution in the flow field are obtained, and the calculation results are shown in Figure 7. The starting position of the particles is located on a circle with a grouting hole as the center and a radius of 0.3 m. The spatial arrangement of particles with the same starting position represents a streamline.

By observing the distribution of streamlines, it is found that the shapes of streamlines with different initial angles are different. The streamline with the initial angle pointing to the downstream diffusion zone always extends along the downstream direction; that is, the horizontal velocity component of gel particles is always greater than zero. The closer the initial angle is to the horizontal streamline, the larger the horizontal velocity component is and the smaller the vertical velocity component is, the faster the gel particles diffuse along the horizontal direction. The streamline in the counter-water diffusion zone first extends a distance in the counter-water direction and then gradually turns to the downstream direction. The closer the initial angle is to the horizontal streamline, the farther the backwater diffusion distance can be. That is, the horizontal velocity component of the gel particles first gradually attenuates from a certain value to zero and then continues to increase. Because the horizontal velocity component of the gel particles first decreases and then increases, and the migration trajectory of the particles is longer, the displacement of the gel particles that initially diffuse to the upstream area after entering the downstream area always lags behind the particles that diffuse to the downstream area at the same initial time. Therefore, the gel particles entering the fracture at the same time gradually form an oval shape in the diffusion process.

**Figure 8:**
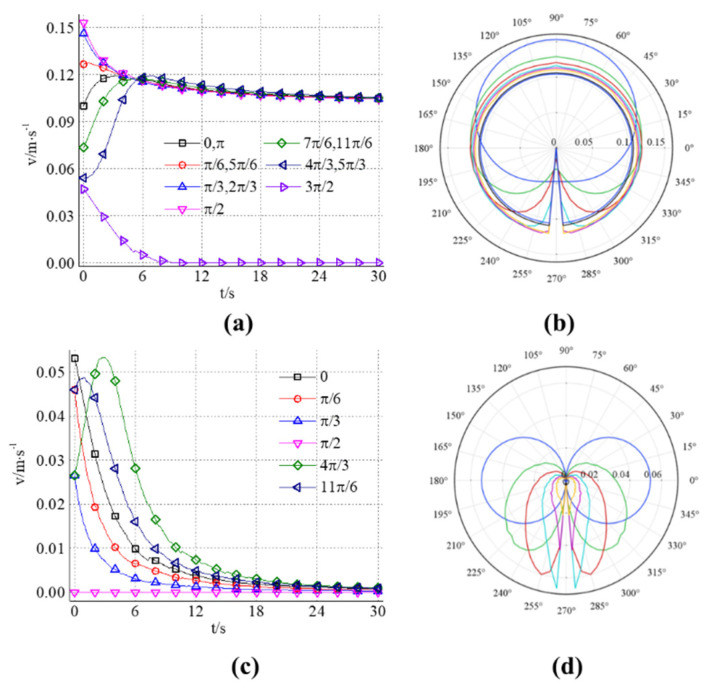
(**a**,**b**) shows the horizontal component of the grout particle velocity changes with the initial angle and grouting time. (**c**,**d**) the vertical component of the grout particle velocity changes with the initial angle and grouting time. Among them, (**a**–**c**) is the change of the streamline velocity with time with different initial angles, and (**b**–**d**) is the change of the velocity of each streamline with the initial angle at different moments.

If the angle between the initial position of the gel particles and the line connecting the grouting hole and the *y*-axis is taken as the parameter, and the clockwise direction is taken as the angle-increasing direction. The relationship curves between the horizontal and vertical velocity components of the streamline with different initial angles and times can be obtained. In Figure 8a, since the streamline distribution is symmetrical about the *x*-axis, the streamline with initial angles of π/6, π/3, 7π/6, and 4π/3, respectively, coincides with the streamline with angles of 5π/6, 2π/3, 11π/6, and 5π/6, respectively. In Figure 8c, the vertical velocity components of the *x*-axis symmetric streamline are equal in value and opposite in sign, so only the streamline distribution with a positive velocity component is shown in the figure. In Figure 8b,d, the graphics connected by the velocity components of different initial angles at any time is a closed curve. In Figure 8b, the grouting time corresponding to the curve from outside to inside on the coordinate line with the initial angle of 90° is 0 s, 2 s, 4 s, 6 s, 8 s, 10 s, 20 s, and 30 s, respectively. In Figure 8d, the times corresponding to the outside-in curve on the 0° and 180° coordinate lines are 0 s, 2 s, 4 s, 6 s, 8 s, 10 s, 20 s, and 30 s, respectively.

It can be seen from Figure 8a that the initial velocity of gel particles corresponding to streamlines with different initial angles is different. When the initial angle is π/2, the initial horizontal velocity component has the maximum value, while the vertical velocity component is zero. It can be seen from Figure 8b that with the increase in grouting time, except for the horizontal velocity component of the streamline with an initial angle of 3π/6, the horizontal velocity component of each streamline gradually approaches 0.1 m/s, that is, the groundwater velocity. The initial velocity of some streamlines with an initial angle between 0 and π is greater than 0.1 m/s and continues to decrease, while the velocity of other streamlines first increases and then decreases. Figure 8b further shows the change trend of velocity distribution with time at different angles. The curve at the initial time is an irregular ellipse, and its centroid deviates greatly from the origin. At this time, the velocity of gel particles is highly asymmetric, and the slurry flow is highly affected by the incident velocity of the grouting hole. With the increase in time, the shape of the curve gradually changes into a circle centered on the origin, which indicates that the influence of the incident velocity of the grouting hole gradually decreases, and the flow of the gel particles is gradually controlled by the flow.

It can be seen from Figure 8c that with the increase in grouting time, the vertical component of velocity in different streamlines eventually decays to zero. When the initial angle of the streamline is in the range of 0 to π/2, the initial value decreases with the increase in the angle and continues to decay with the increase in the grouting time. When the initial angle of streamline is in the range of π/2 to π, the vertical component of particle velocity first increases and then decreases with grouting time and finally approaches zero. By analyzing Figure 8d, it can be further known that the vertical velocity component of the streamline with an initial angle in the range of π to 2π is generally higher than that in the range of 0 to π. With the increase in grouting time, the vertical velocity component of the streamline with an initial angle near π/2 will reach a certain maximum, and the higher the maximum is when the angle is close to π/2, the more it indicates that the vertical diffusion distance of the gel particles on the streamline is larger.

#### 4.1.3. The Effect of Expanding Particles on Fluid Drag

**Figure 9:**
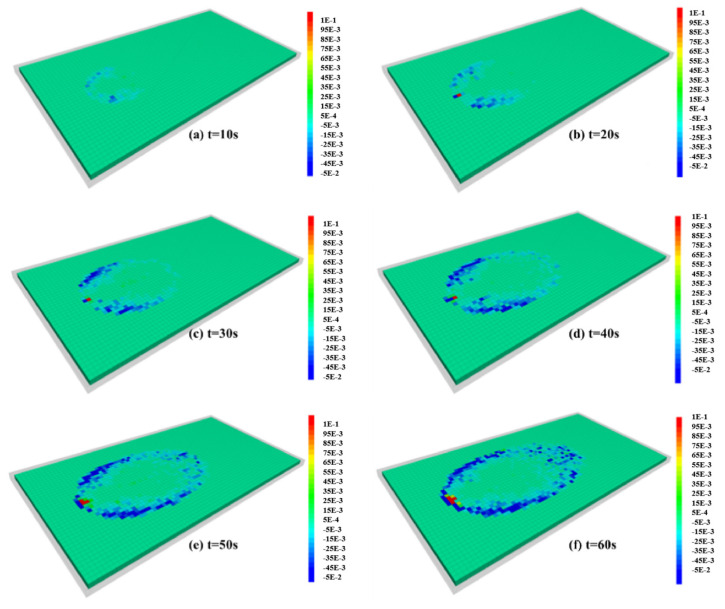
The distribution of the drag force between the gel particles and the fluid in the fracture during the slurry diffusion process: (**a**) t = 10 s, (**b**) t = 20 s, (**c**) t = 30 s, (**d**) t = 40 s, (**e**) t = 50 s, (**f**) t = 60 s.

Figure 9 shows the distribution of drag force between gel particles and fluid in fractures during slurry diffusion. Because the direction of groundwater flow is positive, except for a small part of the area near the grouting hole, the drag force exerted by the gel particles on the fluid is negative, that is, the gel particles hinder the flow of groundwater. In any fluid unit, the drag force of the gel particles on the fluid is determined by the number of particles contained in the fluid unit, the expansion volume, and the velocity difference between the fluid and the gel particles. As the gel particles continue to absorb water and expand, the drag force between particles and fluid will continue to increase. Because the particle expansion affects the flow field distribution in the fracture, and the particle expansion leads to the increase in the interaction between particles and the constant change of their own motion state, the drag force in the fluid unit is not a simple positive correlation with the particle expansion volume but changes with the increase in grouting time and the expansion of slurry diffusion range.

By analyzing the spatial distribution of the drag force, it can be found that the effect of the drag force on the groundwater remains zero beyond the diffusion range of the slurry. In the range of slurry diffusion, the drag force in different fluid units varies greatly due to the expansion volume of gel particles, particle density, and the spatial heterogeneity of the velocity field in fractures. Comparing the spatial distribution of drag force at different times, it can be found that the area with a larger drag force is always concentrated in the periphery of the slurry diffusion area, while the drag force on the fluid in the inner area is always smaller. At the same time, the maximum drag force is distributed in the first half of the perimeter of the slurry diffusion area.

Combining the slurry diffusion morphology (Figure 5) and the gel particle velocity distribution curve (Figure 8) At different times, it can be seen that in this region, the horizontal velocity of the gel particles changes from a negative value to a positive value. Diffusion in the upstream direction rapidly changes to the downstream direction. This shows that there is a strong interaction between gel particles and groundwater, and there is a large velocity difference between them. However, in the second half of the peripheral perimeter, there is almost no direct interaction between the gel particles and the groundwater; the velocity of the gel particles in the horizontal direction has changed from spreading in the counter-water direction to in the along-water direction. With the increase in diffusion distance, the influence of jet velocity on gel particles disappears gradually, and the movement of gel particles is completely controlled by groundwater. At this time, the velocity of gel particles along the horizontal direction gradually approaches groundwater, and the velocity along the vertical direction gradually approaches zero. The gel particles diffuse with the groundwater under the action of inertia. There is almost no velocity difference between them, so the drag force of gel particles on groundwater is very small.

Considering the interaction between the expansion of particle volume and the change of flow field, the “two-fluid model” in the traditional continuum theory cannot analyze the fluid particle interaction in the flow process. The CFD-DEM method adopts the fluid grid method, which can solve the problem of “fluid-solid phase transition” when the finite element software (COMSOL Multiphysics^®^ 5.6) is used alone. Therefore, the CFD-DEM method is more in line with reality, which provides good guidance for the application of grouting and plugging of expandable particulate materials.

### 4.2. Plugging Mechanism of Fracture Grouting in Running Water

#### 4.2.1. Different Types of Slurry Plugging Mechanisms

**Figure 10:**
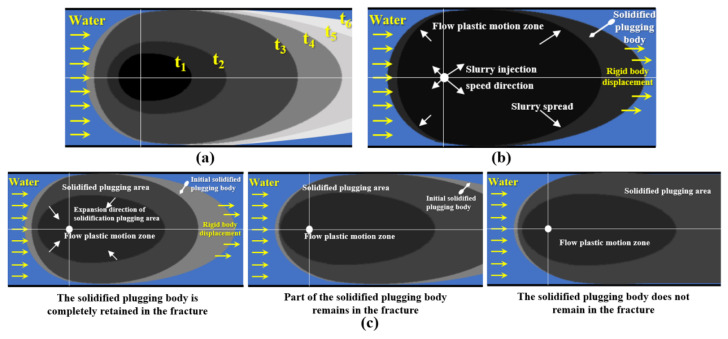
The diffusion process of the cement-sodium silicate slurry in fracture. (**a**) The slurry spreads to the boundary. (**b**) The slurry does not spread to the boundary. (**c**) The three states of the solidified plugging body in fracture.

After the cement-sodium silicate slurry is injected; it follows the asymmetric elliptic diffusion law until it diffuses to the boundary section, as shown in Figure 10a. Then, the slurry diffuses into the pseudo-steady state stage. With the progress of the reaction, the slurry changes into a solidified plugging body, thus completing the dynamic water plugging, as shown in Figure 10b,c. Therefore, the running-water plugging mechanism of cement-sodium silicate slurry under constant grouting factor is essentially the process of slurry diffusion and slurry phase transformation. According to Figure 10, the most favorable time period for the formation of a solidified plugging body is t3–t5. When the formation time of the slurry plugging solidified body is less than t3, the grouting resistance may be too large, and the slurry cannot diffuse to the boundary, so the plugging cannot be formed. When the formation time of the solidified plugging body is greater than or equal to t6, the slurry plugging body will not be able to expand to form the plugging area, and the slurry will be washed out of the rock and soil under the action of groundwater, resulting in waste.

During the grouting process of expandable particulate materials, the slurry migration mechanism is obviously different. Expandable particulate materials mainly rely on the particles continuously absorbing water and expanding to increase their own volume. After the slurry enters the groundwater environment, the carrying liquid is dissolved and diluted rapidly by the groundwater. Therefore, in the process of slurry diffusion, the influence of the physical and chemical properties of the carrier fluid on the micromechanical action and macro migration characteristics of the expandable particles in the groundwater environment can be ignored. Dynamic water grouting can be understood as a process in which the solid particles are continuously injected into the fracture, and then the solid particles and groundwater diffuse together.

The maximum diffusion the opening of gel particles in the diffusion process is the key parameter of dynamic water grouting plugging, which directly determines the plugging area formed after grouting, the residual flow size in the fracture, and the retention rate of grouting materials in the fracture. In the process of grouting, the slurry diffusion opening is affected by the groundwater velocity and grouting rate. When analyzing the slurry diffusion process under the influence of dynamic water, the concept of “slurry water velocity ratio” can be used to characterize the joint influence of groundwater velocity and grouting rate. Its physical meaning is the ratio of grouting flow and the product of groundwater velocity and gap width, as shown in Formula (14).

(14) $$\xi =\frac{Q}{U\cdot d}$$

In the Formula (14), U is the velocity of groundwater, d is the width of the gap, and Q is the grouting flow.

#### 4.2.2. The Effect of Slurry-Water Velocity Ratio on the Diffusion Distance of Expandable Particles

**Figure 11:**
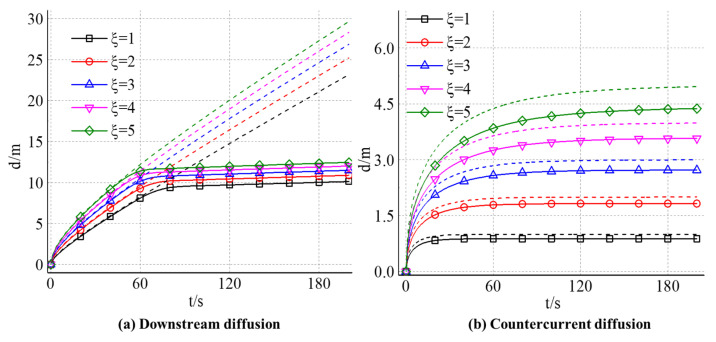
Maximum diffusion distance with different velocity ratio between grout and groundwater (**a**) along the downstream and (**b**) countercurrent direction.

Figure 11 shows the time-dependent variation of slurry diffusion distance along water and counter-water under the influence of different slurry water velocity ratios. The solid line is the actual slurry diffusion distance in the calculation, and the corresponding dotted line is the slurry diffusion distance calculated when ignoring the gel particle volume expansion. Except for the part close to the origin, the curve shows almost linear growth, and the slope of the curve (groundwater) velocity corresponding to different slurry water velocity ratios is the same. At the same grouting time, the larger the slurry-water velocity ratio is, the larger the slurry diffusion distance is. When considering the effect of particle volume expansion, there is an obvious inflection point in the calculated slurry diffusion distance curve. The curve before the inflection point almost coincides with the curve obtained by neglecting the particle volume expansion, which indicates that the gel particle movement is controlled by the flow and grouting rate in the fracture, and the particle volume expansion has little effect on the flow field in the fracture. With the continuous water absorption and expansion of particles, the volume content of particles in the fracture increases, and the interaction strength between particles increases. When the volume fraction of particles is close to 50%, the interaction between particles increases significantly and the resistance to the flow increases rapidly. At this time, the particle migration velocity at the slurry front decreases rapidly, and the curve of diffusion distance forms an inflection point. After the inflection point, the growth rate of the curve decays rapidly and approaches zero, and the slurry diffusion distance hardly increases.

By comparing the slurry diffusion distance curves corresponding to different slurry water speed ratios, with the increase in slurry water speed ratio, the inflection point of the curve is gradually advanced. By observing the curve of slurry diffusion distance with time, it can be found that the curve obtained by considering the effect of particle expansion has a similar growth trend with the curve corresponding to ignoring the effect of particle expansion. The diffusion distance of slurry increases nonlinearly with time, and its growth rate decreases gradually and finally tends to be stable. Compared with the diffusion pattern in the downstream region, there is still a limited diffusion distance in the upstream direction, a stagnation point in the flow field, even without considering the effect of particle volume expansion on the flow. The distance between the stagnation point and the grouting hole is the maximum distance that the slurry can reach along the upstream direction.

The reason for the stagnation point is that the flow field in the fracture can be regarded as the result of the interaction between the parallel flow of groundwater and the point source flow field at the grouting hole. The flow field intensity of a point source decreases with distance, while the field intensity of the parallel flow of groundwater is equal everywhere. In the vicinity of the grouting hole, the flow field strength of the point source is higher than that of the parallel flow field. With the increase in the slurry diffusion distance, the influence of the point source gradually decreases and finally reaches equilibrium, forming a stagnation point in the flow field. By observing the curves corresponding to different slurry water velocity ratios, with its increase, the limit diffusion distance along the upstream direction is increasing, the distance between the stagnation point and the grouting hole is getting farther and farther, and the relationship between them is approximately linear.

When the effect of particle volume expansion is considered; the growth rate of the curve decreases and the limit diffusion distance decreases. Compared with the diffusion distance in the downstream zone, there is no obvious inflection point in the curve, which indicates that the velocity change of gel particles in the upstream diffusion zone is mainly controlled by the flow field in the fracture, and the effect of particle volume expansion on its flow is small. By comparing the curves corresponding to different slurry water velocity ratios, it can be found that the larger the slurry water velocity ratio is, the more obvious the blocking effect of particle volume expansion on flow is and the larger the attenuation range of slurry limiting diffusion distance is.

#### 4.2.3. The Effect of Slurry-Water Velocity Ratio on the Diffusion Velocity of Expandable Particles

**Figure 12:**
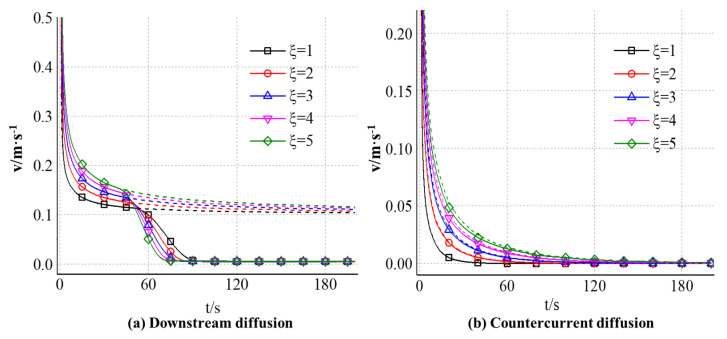
(**a**) The slurry diffusion distance along the downstream and (**b**) countercurrent direction under the influence of different slurry-water velocity ratios.

Figure 12 shows the variation of slurry diffusion velocity with time along the downstream and countercurrent direction of the grouting hole axis under the influence of different slurry-water velocity ratios. The solid line is the actual diffusion velocity of the slurry in the calculation, and the corresponding dotted line is the diffusion velocity of the slurry when the volume expansion of the gel particles is ignored. It can be seen from the analysis in Figure 12a that the diffusion velocity of gel particles along the water direction gradually decreases with time. At the beginning of grouting, when the effect of particle volume expansion is not considered, the diffusion velocity decays logarithmically, and the decay rate decreases gradually. Finally, the diffusion velocity approaches 0.1 m/s (the groundwater velocity). When the effect of particle volume expansion is considered, the trend of velocity change is almost the same at the initial stage of grouting. With the increase in grouting time, the volume of gel particles expands continuously, and the sealing effect of particles on the flow gradually appears. When the grouting time exceeds 40 s, the diffusion velocity begins to decrease obviously. Moreover, the larger the slurry water velocity ratio is, the faster the attenuation rate is and the larger the amplitude is. When the volume of particles in the fracture is large enough, the diffusion velocity will eventually decay to zero.

It can be seen from Figure 12b that the diffusion speed of gel particles along the upstream direction gradually decreases with time. At the initial time of grouting, the diffusion speed of slurry is very fast. With the increase in grouting time, the diffusion distance increases and the decay rate decreases. The curve of velocity change obtained by considering the effect of particle volume expansion is almost the same as that without considering it; the effect of particle volume expansion on its velocity change is small. Compared with the velocity curve in Figure 12a, it can be found that the decay rate of gel particles in the counter-water diffusion zone is faster, and the diffusion rate is more significantly affected by the slurry-water velocity ratio. When the grouting time reaches 30 s, the diffusion rate of gel particles under different slurry-water velocity ratios in the downstream area is maintained between 0.1 m/s and 0.2 m/s, and the maximum difference between different slurry-water velocity ratios is about 30%. However, in the counter-water diffusion region, the diffusion rate has decreased to less than 0.05 m/s, and the diffusion rate corresponding to the slurry-water velocity ratio of 1 is almost zero. When the grouting time reaches 60 s, the particle diffusion rate is still maintained at about 0.1 m/s in the downstream area, while in the upstream area, the diffusion rates corresponding to different slurry-water velocity ratios have declined to zero.

#### 4.2.4. The Effect of Slurry-Water Velocity Ratio on the Opening Degree of Expandable Particles

**Figure 13:**
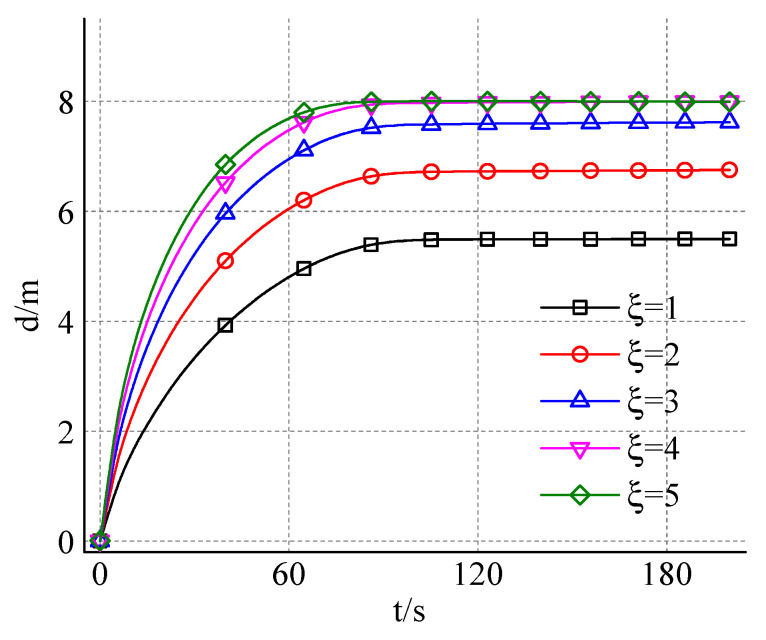
The change of diffusion opening degree with time and different slurry-water velocity ratios.

Figure 13 shows the change of diffusion opening degree with time and different slurry water velocity ratios. It can be seen from Figure 12 that the slurry diffusion opening degree increases nonlinearly with the grouting time, and its growth degree gradually slows down and finally tends to be stable. Combined with the previous analysis, it can be seen that the slurry diffusion shape at any grouting time can be regarded as an irregular ellipse, and the length of its short axis represents the slurry diffusion opening degree at that time. In the initial stage of grouting diffusion, the minor axis of the ellipse is near the grouting hole, and the velocity component of gel particles along the vertical direction is larger, so the growth rate of slurry diffusion opening degree is faster. With the increase in grouting time, the position of the short axis continues to move along the water diffusion direction, the distance between the gel particles and the grouting hole becomes farther, and the volume of the gel particles increases, which leads to the decrease in the velocity component in the vertical direction and the decrease in the growth rate of the slurry diffusion opening. At the same time, because the distance between the gel particles and the boundary on both sides of the fracture gradually shortens, the influence of the fracture boundary on the flow field gradually increases. When the vertical velocity component of the gel particles decreases to zero or the particles diffuse to the side boundary, the slurry diffusion opening degree reaches its maximum. Further analysis of the growth trend of the diffusion opening degree curve shows that when the grouting time exceeds 40 s, the growth rate begins to decrease significantly, and when the grouting time reaches 90 s, the diffusion opening degree basically reaches its maximum. By comparing the diffusion opening degree curves corresponding to different slurry-water velocity ratios, it can be found that with the increase in the slurry-water velocity ratio, the growth rate of the slurry diffusion opening degree increases gradually.

## 5. Conclusions

(1) Based on the CFD-DEM method, a numerical model of grouting diffusion in running water of expandable particulate slurry is established. Compared with the calculation method based on continuum theory, the CFD-DEM method can directly reflect the process of gel particle water absorption and expansion and fully consider the interaction between fluid and suspended particles so as to truly reflect the rheological characteristics of expandable particle slurry.
(2) During the grouting process in running water, the cement slurry initially forms an asymmetric ellipse, later becoming elliptical in the counter-water direction. The downstream diffusion range is significantly greater than that of the counter-water diffusion. In the diffusion of cement-sodium silicate slurry, the traces are asymmetric ellipses and different characteristics will be shown by the boundary of the model. For the expandable particulate slurry, the diffusion is approximately elliptical in the fracture with running water. In the counter-water diffusion region, the migration of the gel particles is mainly affected by flow field control, and the attenuation of its speed is mainly caused by a reduction in slurry pressure. In downstream diffusion, the particle is simultaneously influenced by the flow field and its own volume, and its maximum diffusion distance is determined by both. In grouting engineering, adjusting the grout injection rate and the water absorption rate of gel particles can help control the sealing range.
(3) Cement slurry mainly relies on particle deposition to achieve dynamic water sealing, and the work efficiency is relatively low. Cement-sodium silicate slurry has a rapid solidification effect, which is substantially the process of slurry transition from liquid to solid state. The expandable particulate material is mainly based on gel particles that absorb water into the sealing slurry. For expandable particulate materials, if the sealing area is small after grouting, the remaining gel particles will continue to flow and bypass the agglomeration sealing area. At the same time, the original sealing zone expanded particles may also re-flow underwater erosion, eventually resulting in complete loss of the grouting material and not successfully sealing the flow in the fracture. In grouting engineering, the relationship between the diffusion opening degree and the diffusion distance of the slurry is taken, thereby achieving the best grouting sealing effect.
(4) In the actual hydrodynamic grouting process, the water conduction structure in the formation usually has a more complex geometry, which cannot be reduced to a pipeline with a regular contour or a single slab fracture. The diffusion range and plugging effect of the slurry are significantly affected by the spatial morphology of the water conduction structure. Therefore, it is of great significance to construct a more accurate three-dimensional geological model and establish an accurate and efficient grouting diffusion calculation method on this basis for the theory of hydrodynamic grouting plugging.

## Figures and Tables

**Table 1:** Calculation parameters.

	Fluid Properties		Wall Properties		Particle Properties		
Name	Density	Viscosity	Stiffness	Stiffness	Density	Particle size	Expansion rate
Unit	kg/m^3^	Pa·s	Pa	Pa	kg/m^3^	mm	s^−1^
Value	1000	1 × 10^−3^	1 × 10^9^	1 × 10^3^	1000	1	1

## Data Availability

The original contributions presented in this study are included in the article. Further inquiries can be directed to the corresponding author.
